# The effect of repeated washing of long-lasting insecticide-treated nets (LLINs) on the feeding success and survival rates of *Anopheles gambiae*

**DOI:** 10.1186/1475-2875-9-304

**Published:** 2010-10-29

**Authors:** Francis K Atieli, Stephen O Munga, Ayub V Ofulla, John M Vulule

**Affiliations:** 1 Kenya Medical Research Institute, Centre for Global Health Research, Kisumu, Kenya; 2 Department of Biomedical Sciences and Technology, Maseno University, Kenya

## Abstract

**Background:**

Insecticide-treated nets protect users from mosquito bites, thereby preventing transmissions of mosquito borne pathogens. Repeated washing of nets removes insecticide on the netting rendering them ineffective within a short period. Long-lasting insecticide-treated nets (LLINs) offer longer time protection against such bites because they are more wash resistant, and are preferred to conventionally treated nets. However, there is limited information on the effect of repeated washing of LLINs on the feeding success and survival of wild malaria vectors.

**Methods:**

The current study evaluated the effect of repeated washing of four brands of LLINs on the feeding success and survival rates of *Anopheles gambiae *sl reared from wild strains. In this study, two- to five-day old F1s, reared from gravid mosquitoes collected from an area with a high coverage of LLINs were offered blood meals through protective barriers of the above LLINs. Mosquitoes were exposed for a period of 10 minutes each time. Nets were tested unwashed and subsequently after every 5^th ^through wash 15. After exposure mosquitoes were sorted out according to their feeding status. They were then held under normal laboratory conditions for 24 hours and mortality was scored in both fed and unfed.

**Results:**

It was observed that mosquitoes did not feed through a barrier of unwashed LLINs. However, the feeding success and survival rates increased with successive number of washes and were also net brand dependant. After 15 washes, 49% of vectors succeeded to feed through a protective barrier of PermaNet 2.0 and 50% of the fed died after 24 hrs while after the same number of washes 60% of vectors succeeded to feed through Olyset brand of LLINs and all of them survived. In general, more mosquitoes survived after feeding through Olyset compared to the other four brands that were evaluated. When efficacy of individual LLINs was compared by a t-test analysis to a conventionally treated net, the results were not significantly different statistically for Olyset (*p = *0.239) and NetProtect (TNT) (*p = *0.135). However, the results were highly significant when comparison was made with PermaNet and Interceptor (BASF); *p *values 0.015 and 0.025 respectively.

**Conclusion:**

The result of this study shows that repeated washing of LLINs at short time intervals using local washing methods may render them infective within a short time in preventing local vectors from feeding.

## Background

The use of insecticide-treated nets (ITNs) has been adopted as a standard method for malaria vector control [1]. Long-lasting insecticide-treated nets (LLINs) are recent innovations that have been proven to be more effective and bio-durable and are preferred to conventionally treated nets. Currently, there are several brands of LLINs on the market which have received approval by World Health Organization Pesticide Evaluation Scheme (WHOPES) as LLINs [2]. Among them are four brands, the subject of this study. They include: Olyset^®^, PermaNet^® ^2.0, BASF^® ^and Net Protect^® ^(TNT). Many more others are still under various stages of development. Before receiving WHOPES approval as LLINs, the above nets underwent a standardized testing procedure of undergoing up to more than 20 washes without loosing their effectiveness [3-5].

Effective malaria vector control using LLINs requires strict user compliance by adhering to daily proper deployment, maintenance and replacement of the torn or obsolete nets. The actual useful biological life spans of LLINs under local field conditions are also unknown [6]. The current estimates that are popularly quoted are extrapolations from controlled laboratory studies [7] and a few field studies [8]. More over some of the brands of LLINs on the market such as BASF and TNT are recent innovations that have not been in use for long. In some laboratory studies it has been shown that exposure and repeated washing of LLINs can result in reduction of efficacy due to loss of insecticide [9-11]. It has also been shown in field studies that regular community wide use of one insecticide can result in local vectors developing resistance or tolerance to that particular kind of insecticide [12-14].

The insecticides of choice for net treatment have been pyrethroids based because of their safety profiles on non-target arthropods, low mammalian toxicity and rapid knockdown of targeted vectors [15]. LLINs are now widely accepted as an alternative to ITNs based on laboratory wash bio-durability evidence and on the assumption that laboratory performance can be replicated in the real world in a field situation [8,16]. Given the historical profile of malaria vector control using DDT [16,17] more care must always be taken to evaluate and monitor every behavioral aspect of the targeted vector in order to sustain any control effort. In the absence of such efforts, it is possible that the current vector control measures a lone might not be sustained for long. There is, therefore, a need for constant evaluation and monitoring of new LLIN products as they become widely available and acceptable as malaria vector control tools. Taking such steps will enable malaria control programs in Africa to avoid a situation whereby LLINs are likely to provide only short-term solutions without the need to be complemented by other sustainable tools [18].

The current study was undertaken to evaluate the feeding success and survival rates of wild *Anopheles gambiae *s.l. through four brands of LLINs before and after repeated washing under laboratory conditions. Currently, there is limited information on the effect of repeated washing of LLINs on the feeding success and survival rates of the above vectors.

## Methods

The following 4 brands of LLINs were tested: (i) PermaNet^® ^2.0, a polyester based netting, the insecticide used is deltamethrin @ 55 mg/m^2^, (ii) Interceptor^® ^(BASF) also polyester based netting treated with alphacypermethrin @ 200 mg/m^2^, (iii) Olyset^®^, polyethylene based netting. The insecticide used is permethrin @ 1000 mg/m^2 ^and (iv) NetProtect^® ^polyethylene based netting and the insecticide used is deltamethrin @ 65 mg/m^2^. A conventional polyester based netting treated with deltamethrin @ 25 mg/m^2 ^was used as a control.

### Mosquito feeding procedure

In this study, twenty (20), three (3) day old F1 generations of *An. gambiae *s.l., raised from wild gravid females and larvae collected from an area with high LLINs coverage were released in a larger cage 60 × 60 × 60 cm. A rabbit with its back shaved was placed into a smaller cage measuring 30 × 17 × 15 cm. The shaved part was covered with one of the LLINs under investigation and made to fit into a slit 15 × 10 cm cut at the top of the smaller cage. The smaller cage containing the rabbit was introduced in the bigger cage with mosquitoes making sure that only the shaved and covered part with the relevant brand of LLIN was accessible to the mosquitoes. The feeding success and survival rates of mosquitoes were recorded on each net from each treatment group before washing commenced, and subsequently after wash 5, 10 and 15. For each treatment group and wash cycle, one piece of netting measuring 30 × 30 cm and one rabbit was used. A total of five rabbits were used in the feeding experiments. The rabbits were exposed for a period of 10 minutes to the mosquitoes during each feeding cycle. Each net brand was tested on the 3^rd ^day after the previous washing. The numbers of mosquitoes landing and flying away before feeding during the experimental period were also recorded. At the end of exposure period, mosquitoes were transferred into holding paper cups and scored for number fed and unfed. Mosquitoes in each category were then provided with 5% sugar solution on a moist cotton pad and held at 25 - 27°C and 60 - 80% humidity for 24 hours. Mortality in both groups from each treatment and wash cycle was scored after 24 hours.

### Net washing procedure

Net washing was done using a local washing method of hand rubbing and a local detergent OMO. Washing and drying was done outdoor at KEMRI, Centre for Global Health Research in Kisian village, western Kenya. Four field assistants from the local community were hired to do the washing. Washing was done by immersing the netting in a measured volume of water using a measured detergent. The field assistants were randomly assigned to wash the four brands of nets by hand rubbing. Nets were washed for 10 minutes by immersing each net in two liters of cold rain water mixed with 5 g of detergent. After washing each net was rinsed twice for 5 minutes in same amount of clean water. After washing nets were air-dried by hanging under the shade. Each net was washed twice a week.

## Results

The feeding success and survival rates of mosquitoes exposed to each net brand after repeated washing were recorded (Table 1). It was observed that no mosquito succeeded in feeding through any of the four brands of netting before washing commenced. However, after the 5^th ^washing, the proportion of mosquitoes that succeeded in feeding through the nets was 17.6%, 10.9%, 8.7% and 4% on Olyset, PermaNet, BASF and TNT respectively. After the feeding the survival rates among the fed mosquitoes was monitored 24 hour after exposure. It was observed that 72%, 17%, 33% and 33% of the fed mosquitoes survived after feeding through Oyset, PermaNet, BASF and TNT respectively. The feeding success and survival rates maintained an upward trend with increasing number of washes and after wash 15, 61%, 54%, 44% and 60% succeeded in feeding through Olyset, PermaNet, BASF and TNT respectively. The subsequent survival rates after the above number of washes were, 100%, 53%, 81.8% and 86.7% after feeding through Olyset, PermaNet, BASF and TNT respectively. During the exposure period, mosquito behaviors were also monitored and recorded (Table 2). The number of mosquitoes that landed and flew away before feeding was noted. At baseline before washing commenced, mosquitoes that were exposed appeared highly disturbed and irritated. Some of them attempted to momentarily touch the netting for a few seconds before flying away. Among the four brands of netting that were used in the study, Olyset was more irritating at baseline. The proportion of mosquitoes that attempted to land on each of the nettings before washing were 4%, 28%, 31% and 29 for Olyset, PermaNet, BASF and TNT respectively. All of the mosquitoes that attempted to touch any of the netting and did not succeed in obtaining a blood meal died after 24 hours. After the 5^th ^washing, the landing rates were 18.6%, 10.1%, 9% and 11.2% on Olyset, PermaNet, BASF and TNT respectively. The subsequent mortality rates were 27.8%, 83.3%, 66.7% and 66.7% on Olyset, PermaNet, BASF and TNT respectively. A significant drop in mortality among the unfed mosquitoes was recorded between wash 10 and 15. After wash 15 landing rates recorded were 61%, 49%, 41.9% and 44.3 respectively, and the resultant mortality rates were 2%, 51%, 36.4% and 15.7% on mosquitoes landing on Olyset, PermaNet, BASF and TNT respectively.

**Table 1:** The number of mosquitoes exposed on repeatedly washed LLINs, feeding rates and survival status.

Net Brand	# washes	# Exposed	% Fed	% Dead 24Hrs	% Live 24 Hrs
Conventional	0	104	2 100	0	

Olyset	0	101	0	N/A	0

P/Net	0	100	0	N/A	0

BASF	0	100	0	N/A	0

TNT	0	102	0	N/A	0

Conventional	5	104	11.5	16.7	83.3

Olyset	5	102	17.6	34.4	65.6

P/Net	5	110	10.9	66.7	33.3

BASF	5	103	8.7	66.7	33.3

TNT	5	99	4	75	25

Conventional	10	100	44	11.4	88.6

Olyset	10	100	40	0	100

P/Net	10	110	35	72	27.9

BASF	10	100	28	68	32

TNT	10	105	29	48.3	51.7

Conventional	15	101	82	0	100

Olyset	15	100	61	0	100

P/Net	15	100	54	46.3	53.7

BASF	15	100	44	18.2	81.8

TNT	15	111	60	13.3	86.7

**Table 2:** The total number of mosquitoes exposed, landing rates and their survival status.

Net Brand	# washes	# Exposed	% landed	% dead 24Hrs	% Live 24 Hrs
Conventional	0 X	102	26.5	100	0

Olyset	0 X	101	4	100	0

P/Net	0 X	104	27.9	100	0

BASF	0 X	101	30.7	100	0

TNT	0 X	99	28.3	100	0

Conventional	5 X	103	23.3	20.8	79.2

Olyset	5 X	97	18.6	27.8	72.2

P/Net	5 X	110	10.1	83.3	16.7

BASF	5 X	100	9	66.7	33.3

TNT	5 X	107	11.2	66.7	33.3

Conventional	10 X	101	42.6	14	86

Olyset	10 X	100	42	19.9	80.1

P/Net	10 X	115	34.8	77.5	22.5

BASF	10 X	103	27.2	64.3	35.7

TNT	10 X	100	27	51.5	48.1

Conventional	15 X	100	76	0	100

Olyset	15 X	100	61	2	98

P/Net	15 X	100	49	51	49

BASF	15 X	105	41.9	36.4	63.6

TNT	15 X	115	44.3	15.7	84.3

**Figure 1:**
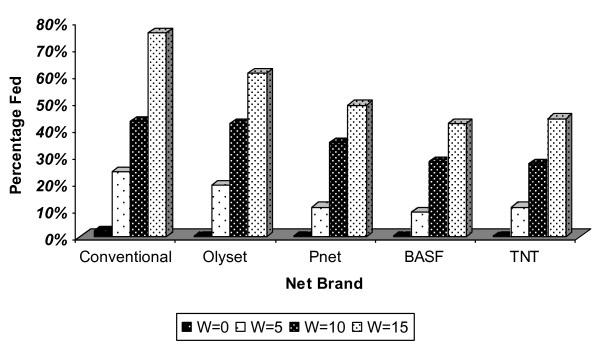
**An. gambiae feeding rates on the four brands of LLINs before and after repeated washing**. shows the percentage of An. gambiae sl that succeeded in feeding on each brand of net after wash 0, 5, 10 and 15 respectively. The nets were hand washed in rain water using a local detergent OMO. Nets were air-dried outdoors under the shade.

The mosquitoes feeding success rate recorded before and after repeated washing on all the four net brands was not uniform (Figure 1). The success rates varied with net brand and by number of washes. In general, as the number of washes increased mosquito feeding success rates also increased on all the four brands of LLINs that were evaluated. The highest feeding success rate was recorded among mosquitoes that fed through Olyset compared to other net brands. For example the success rates were 19 - 61%, 11- 49%, 9 -43% and 11- 44% for that fed through Olyset, PermaNet, BASF and TNT between wash 5 and 15 respectively. The feeding success rates were statistically insignificant (p = 0.78) when compared using ANOVA.

**Figure 2:**
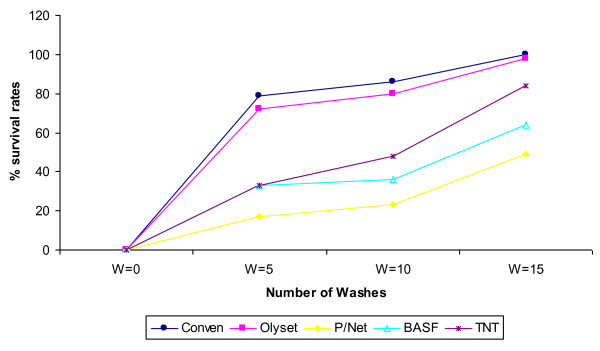
**Survival rates of fed mosquitoes**. shows survival rates of blood fed mosquitoes after repeated washing, 24 hours after exposure.

The mortality rates among the fed mosquitoes were also recorded. It was observed that mortality rates also varied by net brand and by number of washes. Overall the lowest mortality was recoded on Olyset brand of netting while the highest mortality was recorded on PermaNet (Figure 2). At wash 5, mortality rates were 20%, 84%, 67%, and 67% for Olyset, PermaNet, BASF and TNT respectively while at wash 15 it was 2%, 49%, 36% and 16% for Olyset, PermaNet, BASF and TNT respectively.

When the survival rates of vectors 24 hours after feeding through the above LLINs were compared, (Figure 2), it was observed that the survival rates also increased with increasing number of washes and also varied with net brand. The highest number of survivors was on mosquitoes that fed through Olyst brand of netting. At wash 5, 72% of those that fed through this brand of netting survived while at wash 15, 98% survived. For those vectors that fed through other three brands of LLINs, PermaNet recorded 16% and 49%, BASF, 33% and 64% and TNT, 33% and 84% at 5 washes and 15 washes respectively.

**Figure 3:**
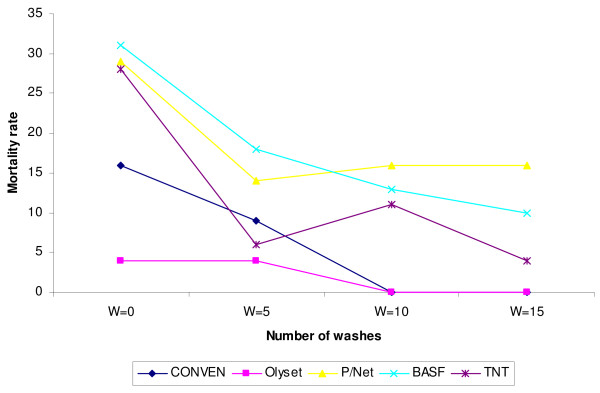
**Mortality rates among the unfed mosquitoes**. shows the mortality rates among the mosquitoes that did not succeed in obtaining a blood meal through the four brands of LLINs after 24 hrs post exposure.

The mortality rates of mosquitoes that came into contact with the nets at short intervals in an effort to get a blood meal and were irritated and flew away before feeding was recorded (Figure 3). It was observed that the short contact resulted in a significant mortality among the vectors that did not feed. Overall the mortality rates among the unfed mosquitoes were much lower than the survivors. For Olyset it was 16% and 0%, for PermaNet, mortality was 29% at baseline and 16% at wash 15 for BASF it was between 35% and 10% and for TNT it was 28% to 4% at baseline and wash 15 respectively.

In general, it was observed that the Olyset net brand was the least effective among the net brands that were evaluated. When its efficacy was individually compared by a paired t-test analysis to a conventionally treated net and TNT, a net brand with similar netting material, the difference in efficacy were not statistically significant. The p values were 0.239 and 0.135 respectively. The results were highly significant when this net was compared to PermaNet and BASF with p values of 0.015 and 0.025 respectively.

## Discussion

The current study evaluated the feeding success and survival rates of *An. gambiae *through four brands of LLINs namely Olyset, PermaNet BASF and TNT before and after repeated washing under laboratory condition. The study has shown that the LLINs which were evaluated were very effective in preventing mosquitoes from feeding through them before washing. However, as the washing progressed it was observed that the number of mosquitoes feeding gradually increased. The increase in feeding and survival rates with subsequent washing was net brand dependant. The study also observed that among the four brands of LLINs evaluated the feeding rates and survival after feeding were lower among the mosquitoes that fed through PermaNet and were highest among the mosquitoes that fed through Olyset, compared to other brands of LLINs.

There are no studies directly comparing the feeding and survival success of *An. gambiae *on the above brands of LLINs. However, there are related studies, which have evaluated the effect of ITNs on blood feeding and house exit behavior. In one such study Mathenge and others [19] evaluated the effect of permethrin-impregnated nets on exiting, blood feeding success, and time of feeding of malaria vectors in western Kenya. The study found that the number of *An. gambiae *s.l. entering houses was unaffected by the presence of bed nets, but *An. gambiae *s.s. and *Anopheles funestus *were more likely to exit bed-netted houses. The study also found that although there was a small shift in biting time, no differences were detected in *Anopheles arabiensis *rates of blood feeding and exiting. The current study seems to confirm that *An. gambiae *s.l. has the ability to adapt to some extend to the presence of insecticide-treated nets. The repellency effect that was observed which deterred field mosquitoes from landing and feeding was strongest before washing and appeared to diminish with repeated washing, hence the observed increase in feeding and survival rates with subsequent increase in the number of washes. This study also observed that all the LLINs evaluated were equally effective in preventing mosquitoes from feeding when unwashed. In general, repeated washing resulted in reduced mortality and an increase in survival rate. It is important to note that nets were not washed according to WHOPES protocol which uses specific soap and machine. The washing procedure and soap used in the current study is similar to the one commonly used in the local villages in rural areas. The short time interval between washes might not have accorded enough time for polyethylene based LLINs to regenerate, hence might have resulted in the observed low feeding deterrent rates observed on Olyset nets. However, in a field situation where people live and cook in single roomed houses nets accumulate dirt from soot and over-handling within a short time which often results in increase in washing frequency. The observed increase in the feeding success and survival rates also varied with net brand. The highest mortality was recorded on vectors that fed through PermaNet, and the least mortality was recorded on mosquitoes that fed on Olyset. Polyester based LLINs might have performed better than Polyethylene based LLINs due to shorter time interval required for regeneration. Olyset has been reported to take up to 15 days for self regeneration to occur [20], but the self regeneration period of this net is still controversial. Elsewhere after the same period of time, Olyset nets that were held at 30°C did not regenerate [7]. The current study raises an important question on what might happen in a field situation between washes of such kind of netting. Even if the washing interval is increased to allow for the regeneration, sleepers will be exposed to field mosquitoes within the regeneration period.

The results of the current study have shown that there could be a relationship between the progressive increase in feeding success and reduction in mortality with successive increase in the number of washes. This finding concurs with the findings of other studies which have reported that the effectiveness of LLINs is affected by the number of washes. In one laboratory based study carried out at CDC in Atlanta USA, comparing wash resistant of six types of LLINs, mortality of less than 10% was recorded on Olyset brand of LLIN after only six washes using susceptible laboratory reared *An. gambiae *s.s. in cone bioassay tests [7]. The current study found that among the four brands of LLINs evaluated, PermaNet was more effective. These finding concurs with another related study carried out in Iran, which compared the bio-efficacy of three brands of LLINs PermaNet, Yorkool and A-Z nets. The study found that PermaNet was more wash resistant and bio-effective [9]. Elsewhere in a related study also carried out in Iran, evaluating the effect of washing on the bio-efficacy of Olyset using cone bioassays, Rafinejad and others observed a 97% mortality on unwashed Olyset nets and 9% mortality after 20 washes [18]. In the current study, it was observed that more vectors succeeded in feeding through Olyset compared to BASF and TNT and those that attempted to feed through PermaNet were least successful.

Among the four LLINs, which were evaluated, Olyset and PermaNet have been on the market longest and are the most studied. There is an accumulation of field use data on the above two brands which has enabled them utilize the feed back for product improvement. PermaNet for example has evolved from PermaNet 1.0 to 2.0 and it is still undergoing improvement. However, most of the studies carried out comparing the bio-efficacy of the two markets dominated LLINs [7-9] have consistently shown that Olyset is more wash durable and less bio-effective compared to PermaNet may be because of the treatment technology used and netting material [15]. The current study adds more evidence on the findings of the above earlier studies. Repeated washing generally affected the ability of all the LLINs to prevent mosquitoes from feeding. It is also evident that Olyset with its superior wash durability was least effective in killing and preventing mosquitoes from feeding may be because of the longer time required for insecticide to migrate from the inside of the fibres to the net surface. The current study was conducted under laboratory condition using adult mosquitoes reared from larvae and F1 generation of field collected mosquitoes from an area where PermaNet and Olyset brands of LLINs have been in use for a period of over two years. Based on these findings, it is necessary that this kind of study be expanded and carried out in a field setting using free-flying wild mosquitoes

The expected protection by LLINs against malaria vectors is based on the assumption that the products will remain effective for a longer time, killing or repelling mosquitoes in a real world situation regardless of the washing methods used. WHOPES set criteria for LLINs approval [2,3], is only a guideline and cannot be expected to be applicable in a field situation. Based on WHOPES recommendation, it is generally believed that washing of LLINs below 20 washes has no effect on their efficacy. These assumptions are sustained by the fact that laboratory studies using susceptible strain of mosquitoes have repeatedly shown that LLINs offer long time protection [8]. More over, previous studies had shown that sleeping under untreated net offers limited protection because when a sleeper comes in contact with the untreated netting mosquitoes can bite through. The introduction of ITNs was an improvement because they offered both physical and chemical barrier [20]. The challenges that were encountered then were the unscheduled frequent washing by users to keep nets clean unaware of insecticide loses and re-treatment compliance was very low [21]. The introduction of LLINs was to enable users wash their nets as needed without compromising their effectiveness. In this regard LLINs had a triple effect of combining physical and chemical barriers to washing durability of the insecticide [3]. This study has shown that the short frequency used in washing LLINs using a local method and detergent, could cause the protective efficacy against wild mosquitoes to diminish and therefore should be avoided.

Given the current finding, it might be feasible to reduce malaria cases by half as projected at the Africa summit meeting in 2000 on Roll Back Malaria [22], by using LLINs in focal groups, but it might not be possible to reduce these cases beyond the above projections, even though universal net coverage and use in malaria endemic zone of western Kenya is a real possibility [23]. Currenly, LLINs are abundantly available at subsidized prices in government and public health facilities [24]. Before the adoption of LLINs, impressive results had been achieved in lowering malaria cases using ITNs. For example, in one control program conducted in western Kenya, Ter Kuile *et al *associated a 60% reduction in both clinical malaria and severe anaemia to ITNs use [25]. In a similar study also conducted in western Kenya, Phillips-Howard *et al *observed a 23% protective efficacy of ITNs for children under 5 years [26]. In the same study, a 59% drop in entomological indices and sporozoite rate was associated with ITNs use in western Kenya [27]. In all the above studies, ITN coverage and use was under strict supervision. But it is curious to note that reduction in malaria cases did not correspond to the massive net coverage. Similar results were also recorded in Ghana, and Tanzania, [28,29] among other sub-Sahara African countries. Because of the above successes, it was projected that the introduction of LLINs will consolidated gains that had been made and drive further down malaria transmission rates to bellow national disease burden levels among developing countries in sub-Sahara African countries like Kenya [30]. To date this has not happened six years after official adoption of LLINs and malaria is still the leading cause of infant and childhood mortality [2].

From the current study, it can be speculated that there are other complex factors related to vector behavior that are not fully understood. Mosquitoes can quickly adapt to the presence of treated nets by momentarily avoiding contact when nets are freshly introduced but quickly rebound in numbers and start feeding once washing of the nets starts taking place. Validation of efficacy of new LLINs products is derived from bioassay data of directly exposing susceptible vectors to the nets without the alterative of offering them a chance to obtain a blood meal and then assessing their survival rates. The current study attempted this approach. This approach was used based on published evidence that vectors have inherent intrinsic behavior of circumventing lethal exposures to insecticide-treated materials, thereby perpetuating malaria transmission at low levels and maintaining their survival [12-14]. This can also be explained from the fact that village-wide coverage does not immediately translate into interrupting the vector human contact circle sufficiently enough to stop transmission [25,26]. Indeed from studies conducted in western Kenya using ITNs, it was observed that despite mass distribution and evidence of daily use, which were strictly monitored by research assistants, transmission still occurred. Various explanations were then advanced. One of them was that vectors were biting earlier before people went to bed [31]. But evidence has also been shown of community wide mass effect of treated nets [32,33]. The observations reported by the current study possess challenges to malaria control programs of what might be happening in the filed. If wild vectors can feed through washed LLINs because of the shortened interval between washes or longer periods required after washing for some LLINs to regenerate, then it might be time for vector control experts to think on stop gap measures to use for protecting LLIN users between the regeneration periods. This might be a challenge to the whole strategy of using LLINs as a key component of controlling malaria vectors.

## Conclusions

The evidence from the current study of wild vectors feeding through protective barriers of washed LLINs and subsequent increase in survival rates with progressive washing is of major concern. This evidence although not conclusive, suggests that the incorporation/coating technology used on the netting needs to be improved to achieve better performance. Greater attention needs to be given on regeneration time interval that the insecticide takes to migrate from the inside of the fibres to net surface. LLINs distribution should be accompanied by education on proper usage, washing frequency and interval.

The observation that vectors which succeeded in feeding through washed LLINs had higher survival rates needs further investigation to determine the fate of those vectors in a field situation. With increasing availability of LLINs in areas with abundant vector population such as western Kenya, large population of vectors that might survive sub-lethal exposures doses are likely to replace the susceptible population and pose a serious health risk.

## Competing interests

The authors declare that they have no competing interests.

## Authors' contributions

FKA, conceived designed the study and participated in data collection analysis and writing of the manuscript. SOM participated in data analysis and writing of the manuscript. AVO provided general guidance in proposal development and participated in data analysis and writing of the manuscript. JMV participated in proposal development and provided genera guidance in data analysis and drafting of the manuscript. All authors have read and approved the final manuscript.
